# Associations between the Mediterranean lifestyle and incident age-related eye diseases: a longitudinal analysis from the UK Biobank

**DOI:** 10.7189/jogh.16.04015

**Published:** 2026-01-12

**Authors:** Yi Li, Yuzhou Zhang, Gavin Wong, Ka Wai Kam, Mary Ho, Sunny Au, Xiu Juan Zhang, Mandy PH Ng, Patrick Ip, Alvin L Young, Chi Pui Pang, Clement C Tham, Li Jia Chen, Jason C Yam

**Affiliations:** 1 Department of Ophthalmology and Visual Sciences, The Chinese University of Hong Kong, Hong Kong SAR, China; 2 Joint Shantou International Eye Centre of Shantou University and The Chinese University of Hong Kong, Shantou, China; 3 Hong Kong Eye Hospital, Hong Kong SAR, China; 4 Department of Ophthalmology and Visual Sciences, Prince of Wales Hospital, Hong Kong SAR, China; 5 Hong Kong Hub of Paediatric Excellence, The Chinese University of Hong Kong, Hong Kong SAR, China; 6 Department of Ophthalmology, Hong Kong Children’s Hospital, Hong Kong SAR, China; 7 Department of Ophthalmology, Tung Wah Eastern Hospital, Hong Kong SAR, China; 8 Department of Ophthalmology, The University of Hong Kong, Hong Kong SAR, China; 9 Department of Paediatrics and Adolescent Medicine, The University of Hong Kong, Hong Kong SAR, China

## Abstract

**Background:**

The Mediterranean lifestyle (MEDLIFE) is generally considered to have a positive effect on several health outcomes. However, little is known about its impact on age-related eye diseases. We aimed to assess the effect of MEDLIFE on the risk of three such diseases: cataract, glaucoma, and age-related macular degeneration (AMD).

**Methods:**

We included 113 829 participants from the UK Biobank who were free of age-related eye diseases at baseline and followed them up prospectively for disease occurrence. Adherence to MEDLIFE was assessed using 25 items, categorised under three blocks: ‘Mediterranean food consumption’ (block 1), ‘Mediterranean dietary habits’ (block 2), and ‘physical activity, rest, social habits’ (block 3). We used a Cox proportional hazard model to examine the associations of the MEDLIFE index and each of its blocks with incident age-related eye diseases.

**Results:**

During a median follow-up of 10.5 years, 9954 cases of cataract, 1956 cases of glaucoma, and 1736 cases of AMD were identified. We noted an inverse association between the MEDLIFE index and new-onset cataract (*P*-value for trend = 0.005). A one-point increment in the MEDLIFE score was associated with a 1.5% (95% confidence interval (CI) = 0.7–2.3) reduction in the risk of cataract, and with a 2.4% (95% CI = 0.5–4.3) reduction in AMD incidence. Analysis of MEDLIFE blocks indicated that block 2 (hazard ratio (HR) = 0.97; 95% CI = 0.95–0.99) and block 3 (HR = 0.97; 95% CI = 0.95–0.99) were associated with lower risk of cataract. Block 2 was further related to reduced risk of AMD (HR = 0.95; 95% CI = 0.91–0.99). Although we found no association between the MEDLIFE index and incident glaucoma, block 3 was associated with lower glaucoma risk (HR = 0.94; 95% CI = 0.90–0.98).

**Conclusions:**

Higher adherence to MEDLIFE was associated with decreased incidence of cataract and AMD, while the ‘physical activity, rest, and social interactions’ block was related to a lower risk of glaucoma. Our findings suggest that MEDLIFE may serve as a potential behavioural intervention for preventing age-related eye diseases.

Worldwide improvements in socioeconomic status and longer life expectancies have led to a rise in the number of individuals living with blinding ophthalmic conditions and an increase in their average age. Among these, cataract, glaucoma, and age-related macular degeneration (AMD) stand as the principal causes of blindness and visual impairment globally, especially among individuals aged ≥50 years [1,2]. This growing burden is expected to pose substantial healthcare and economic challenges for both individuals and society, highlighting a need to focus on primary prevention by identifying modifiable risk factors for these conditions.

In this sense, it has been recognised that, alongside pharmacological and surgical treatments for age-related eye diseases, lifestyle interventions are emerging as cost-effective methods for eye care and should be a strong focus in patient care [3,4]. Several lifestyle factors, such as diet, physical activity, and smoking, have been suggested to play a role in the pathogenesis of ageing [5–8], while prior research has indicated that lifestyle modifications can influence the risk of developing age-related eye diseases [9–12]. However, it is currently unclear whether combining multiple lifestyle factors, such as diet, culinary activities, and physical activity, can strengthen their preventive effect for age-related eye diseases. In this context, the Mediterranean lifestyle (MEDLIFE) [13,14], also known as the new Mediterranean Diet Pyramid, comprises several distinctive habits in addition to the Mediterranean diet, such as culinary activities, physical activity, adequate rest, and social activities. Epidemiological findings have suggested that higher adherence to the MEDLIFE is associated with reduced risk for depression, metabolic syndrome, frailty, and all-cause mortality in different populations [15–20], likely due to the synergistic effects on systemic inflammation, oxidative stress, and weight maintenance stemming from the various components of this specific lifestyle pattern.

Studies have also suggested an association between the Mediterranean diet and a lower risk of progression to advanced AMD [21,22]. However, considering the limited knowledge on the potential impact of a wider MEDLIFE on the prevention of age-related eye diseases, we aimed to investigate the association between adherence to the MEDLIFE and the occurrence of cataract, glaucoma, and AMD.

## METHODS

### Study participants

The UK Biobank baseline survey (2006–10) recruited 502 248 participants aged 40–69 years through 22 assessment centres across England, Scotland, and Wales [23]. Their sociodemographic, lifestyle, health, and physical assessment data were collected through touchscreen questionnaires and physical measurements. 

The UK Biobank received ethical approval from the North West Multi-Centre Research Ethics Committee. All participants provided informed consent through electronic signature at the baseline assessment. The anonymised data used in this study is available in the UK Biobank database under application number 91320. Our analysis aligns with the Journal of Global Health’s Guidelines for Reporting Analyses of Big Data Repositories Open to the Public (Table S10 in the **Online Supplementary Document**)

### Assessment of MEDLIFE

We obtained lifestyle data from the UK Biobank touchscreen questionnaires. Specifically, dietary data were collected at recruitment using the Oxford WebQ questionnaire, which gathered information on the quantities of 206 types of foods and 32 types of drinks consumed over the previous 24 hours [24]. A sub-cohort of the UK Biobank completed the assessment one to five times between April 2009 and June 2012. To collect typical dietary intake, we only included data from participants who had completed at least two dietary assessments and used the average food/nutrient intakes of two or more dietary assessments in the analysis. Food intakes were estimated from the frequency of intake of each food or beverage, multiplied by the standard portion sizes. Nutrient intake was calculated by multiplying the amount consumed by the nutrient composition values based on the UK Nutrient Databank Food Composition Tables [25].

The adapted MEDLIFE index, tailored for use in the UK Biobank study, was developed based on insights from previous studies [15,20]. Here we adapted the MEDLIFE index to include 25 out of 29 items due to the unavailability of information on the consumption of olive oil, sofrito (a traditional sauce with olive oil, tomato, and garlic), nibbling outside meals, and eating in company within the UK Biobank data set (Table S1 in the **Online Supplementary Document**). The MEDLIFE index consists of three blocks: ‘Mediterranean food consumption’, with 12 items about food intake (*e.g.* sweets, vegetables, white meat, fruits, fish); ‘Mediterranean dietary habits’, with seven items on habits and practices around meals (*e.g.* low salt consumption, limit sugar-sweetened beverages); ‘Physical activity, rest, social habits and conviviality’, with six items about physical activity and collective activities (*e.g.* physical activity, adequate sleep, collective sports, socialising with friends). Each lifestyle factor is scored 1 if person adhered to it, or 0 points otherwise; the total MEDLIFE score thus ranged from 0 to 25, with a higher score indicating a higher level of adherence.

### Ascertainment of outcomes

The presence of a disease at baseline was determined either by participants’ self-reporting of a doctor’s diagnosis or through inpatient records. Incident cases of cataract, glaucoma, and AMD were identified by their corresponding International Classification of Diseases, 10th Revision codes in medical records: H25, H26, or H28; H40 or H42; and H35.3, respectively [26]. At the time of analysis, hospital admission data were available for participants until 31 October 2022. Participants with cataract, glaucoma, and AMD at baseline were excluded from the analysis for each disease. Individuals were considered at risk from the date of the most recently completed dietary questionnaire, until the date of the first occurrence of age-related eye diseases, date of death, date lost to follow-up, or end of available follow-up (31 October 2022), whichever occurred first.

### Assessment of covariates

Covariates included age, sex, ethnicity, education level (college or university degree, or others), assessment centre, Townsend Deprivation Index (TDI), smoking status (current smoker, previous smoker, or non-smoker), total energy intake, body mass index (BMI) categories (underweight, normal weight, overweight, or obese), and history of hypertension or diabetes, all of which were collected at baseline. The TDI used census data on employment, housing, and social class based on the postal code of participants. The BMI was calculated as weight (in kg)/height (in m)^2^, with weight and height measured at the baseline visit. Total energy intake (continuous, in kcal/d) was calculated from the WebQ [27]. History of hypertension and diabetes was recorded from self-reported diagnoses, medication use, and hospital admission data.

### Statistical analysis

We presented baseline characteristics by quartiles of the MEDLIFE index as means and standard deviations (SDs) and numbers and percentages for continuous and categorical variables, respectively. We estimated hazard ratios (HRs) and 95% confidence intervals (CIs) using the Cox proportional hazards regression models to assess the association between the MEDLIFE index and the risk of each incident the three age-related eye diseases. We established three models: model 1 included adjustment for age, sex, ethnicity, TDI, assessment centre, education level, and smoking status; model 2 was additionally adjusted for BMI categories and total energy intake; while model 3 was further adjusted for history of hypertension and diabetes. We tested the proportional hazards assumption by Schoenfeld residuals (*P* > 0.05 for all Schoenfeld global tests), and all tested hypotheses were found to be valid.

We carried out further subgroup analyses by age (<60, ≥60 years), sex (male, female), smoking status (current smoker, previous smoker, non-smoker), and BMI (<25, ≥25). The modification effect was tested using likelihood-ratio tests, which compared models with and without a cross-product interaction term of MEDLIFE score and the stratified variables. We also conducted sensitivity analyses to verify the robustness of our results. First, we restricted the analysis to participants with at least three diet assessments to explore potential deviations from the usual dietary patterns. Second, to minimise the effect of reverse causation, we additionally excluded participants with disease events in the first two years of follow-up [28–30]. Third, we employed two surrogates (olive oil consumption and tomato-based sauce consumption) from the UK Biobank to estimate the score for olive oil intake and sofrito intake, in accordance with previous studies [31,32], which enabled us to derive a more comprehensive MEDLIFE score, comprising 27 items. Fourth, we focused exclusively on participants from white populations.

We conducted all analyses using SPSS, version 27.0 (IBM Corp., Armonk, NY, USA) and *R*, version 4.4.2 (R Core Team, Vienna, Austria). A two-tailed *P* < 0.05 was considered significant.

## RESULTS

Out of 126 775 individuals within the UK Biobank with at least two 24-hour dietary assessments, we excluded 64 participants without more than two MEDLIFE items, 873 without sociodemographic data, 220 without smoking status data, 266 without body mass index (BMI) data, 144 with missing comorbidity information, and 2503 with extreme energy intake at baseline. We further excluded 8876 participants with any prevalent age-related eye diseases. This left us with 113 829 participants for analysis (Figure S1 in the **Online Supplementary Document**).

### Baseline characteristics of participants

At baseline, the mean age of the 113 829 participants was 55.8 (SD = 7.8) years; 50020 (43.9%) were men, and 110506 (97.1%) were white. The average MEDLIFE index was 9.5 (SD = 2.5) points. Participants with higher adherence to the MEDLIFE were predominantly older, women, non-smokers, had higher educational qualifications, had lower BMI, and had a lower prevalence of hypertension and diabetes compared with those with lower adherence (**Table 1**).

**Table 1:** Baseline characteristics of the UK biobank participants by quartiles of MEDLIFE score

	Quartile 1, 0–7 p (n = 24 685)	Quartile 2, 8–9 p (n = 33 649)	Quartile 3, 10–11 p (n = 31 226)	Quartile 4, 12–25 p (n = 24 269)	Total (n = 113 829)
**Sex, male**	12 501 (50.6)	15 492 (46.0)	13 172 (42.2)	8855 (36.5)	50 020 (43.9)
**Age in years, x̄ (SD)**	54.8 (8.0)	55.6 (7.8)	56.2 (7.7)	56.4 (7.6)	55.8 (7.8)
**Ethnicity**					
White	23 984 (97.2)	32 714 (97.2)	30 263 (96.9)	23 545 (97.0)	110 506 (97.1)
Non-white	701 (2.8)	935 (2.8)	963 (3.1)	724 (3.0)	3323 (2.9)
**Region of assessment**					
England	22 454 (91.0)	30 762 (91.4)	28 562 (91.5)	22 385 (92.2)	104 163 (91.5)
Wales	835 (3.4)	1095 (3.3)	947 (3.0)	693 (2.9)	3570 (3.1)
Scotland	1396 (5.6)	1792 (5.3)	1717 (5.5)	1191 (4.9)	6096 (5.4)
**Education level**					
College or university degree	9882 (40.0)	15 323 (45.5)	15 467 (49.5)	13 277 (54.7)	53 949 (47.4)
Others	14 803 (60.0)	18 326 (54.5)	15 759 (50.5)	10 992 (45.3)	59 880 (52.6)
**Deprivation index**	−1.6 (2.9)	−1.7 (2.8)	−1.7 (2.8)	−1.7 (2.8)	−1.7 (2.8)
**Smoking status**					
Never	13 461 (54.5)	19 188 (57.0)	18 362 (58.8)	14 544 (59.9)	65 555 (57.6)
Former	8740 (35.4)	11 998 (35.7)	11 069 (35.5)	8630 (35.6)	40 437 (35.5)
Current	2484 (10.1)	2463 (7.3)	1795 (5.7)	1095 (4.5)	7837 (6.9)
**Total energy intake in kj/d, x̄ (SD)**	9110.1 (2002.5)	8701.6 (1952.9)	8450.9 (1914.0)	8200.4 (1873.7)	86 143.6 (1962.1)
**BMI categories**					
Underweight (<18.5)	97 (0.4)	167 (0.5)	170 (0.5)	215 (0.9)	649 (0.6)
Normal weight (18.5–24.9)	7616 (30.9)	12 282 (36.5)	12 985 (41.6)	11 884 (49.0)	44 767 (39.3)
Overweight (25–29.9)	10 397 (42.1)	14 142 (42.0)	12 820 (41.1)	9175 (37.8)	46 534 (40.9)
Obese (≥30)	6575 (26.6)	7058 (21.0)	5251 (16.8)	2995 (12.3)	21 879 (19.2)
**Hypertension**	6680 (27.1)	8584 (25.5)	7453 (23.9)	5303 (21.9)	28 020 (24.6)
**Diabetes**	1228 (5.0)	1326 (3.9)	1059 (3.4)	669 (2.8)	4282 (3.8)
**MEDLIFE index, 0–25 p, x̄ (SD)**†	6.2 (1.0)	8.5 (0.5)	10.5 (0.5)	13.1 (1.3)	9.5 (2.5)
Block 1: Mediterranean food consumption, 0–12 p	2.1 (1.1)	3.2 (1.1)	4.1 (1.2)	5.5 (1.4)	3.7 (1.7)
Block 2: Mediterranean eating habits, 0–7 p	2.3 (1.0)	3.0 (1.0)	3.5 (1.1)	4.1 (1.1)	3.2 (1.2)
Block 3: physical activity, rest, social habits, and conviviality, 0–6 p	1.7 (0.9)	2.3 (1.0)	2.8 (1.0)	3.4 (1.1)	2.6 (1.2)

BMI – body mass index, MEDLIFE – Mediterranean lifestyle, SD – standard deviation, x̄ – mean

*Values are presented as n (%) unless specified otherwise.

†Higher indexes indicate higher adherence to MEDLIFE.

### Association between MEDLIFE and risk of age-related eye diseases

During a median follow-up duration of 10.5 (interquartile range = 10.4–10.9) years, 9954 (8.7%) new cases of cataract, 1956 (1.7%) new cases of glaucoma, and 1736 (1.5%) new cases of AMD were identified. We found an inverse association between the MEDLIFE index with new-onset cataract (*P*-value for trend = 0.005). Accordingly, participants in the second (HR = 0.93; 95% CI = 0.88–0.98), third (HR = 0.92; 95% CI = 0.87–0.98), and fourth quartile of MEDLIFE (HR = 0.91; 95% CI = 0.86–0.97) had a significantly lower risk for new-onset cataract, compared with those in the first quartile. For AMD, compared to individuals with the lowest MEDLIFE quartile, those in the highest quartile had a 15% reduced risk of developing AMD (HR = 0.85; 95% CI = 0.73–0.99). A one point increment in the MEDLIFE score was associated with a 1.5% (95% CI = 0.7–2.3, *P* < 0.001) reduction in the risk of developing cataract, and a 2.4% (95%CI = 0.5–4.3, *P* = 0.015) reduction in the incidence of AMD. There was no significant association between the MEDLIFE index and new-onset glaucoma (**Table 2**).

**Table 2:** Association between the MEDLIFE index and risk of age-related eye diseases in the UK biobank*

	Per one point increase		Quartile 1 (0–7 p)	Quartile 2 (8–9 p)		Quartile 3 (10–11 p)		Quartile 4 (12–25 p)		*P*-value for trend
	**HR (95%CI)**	***P*-value**		**HR (95%CI)**	***P*-value**	**HR (95%CI)**	***P*-value**	**HR (95%CI)**	***P*-value**	
**Cataract**										
Events, n/N			2102/24 685	2855/33 649		2805/31 226		2192/24 269		9954/113 829
Model 1	0.98 (0.97–0.99)	<0.001	ref	0.92 (0.87–0.97)	0.002	0.90 (0.85–0.95)	<0.001	0.88 (0.83–0.93)	<0.001	<0.001
Model 2	0.98 (0.98–0.99)	<0.001	ref	0.92 (0.87–0.98)	0.007	0.92 (0.86–0.97)	0.003	0.90 (0.85–0.96)	0.001	0.002
Model 3	0.98 (0.98–0.99)	<0.001	ref	0.93 (0.88–0.98)	0.011	0.92 (0.87–0.98)	0.006	0.91 (0.86–0.97)	0.003	0.005
**Glaucoma**										
Events, n/N			416/24 685	567/33 649		556/31 266		417/24 269		1956/113 829
Model 1	0.99 (0.97–1.01)	0.23	ref	0.94 (0.83–1.07)	0.36	0.95 (0.84–1.08)	0.42	0.90 (0.78–1.03)	0.13	0.17
Model 2	0.99 (0.97–1.01)	0.30	ref	0.95 (0.83–1.08)	0.58	0.96 (0.84–1.09)	0.51	0.91 (0.79–1.04)	0.17	0.22
Model 3	0.99 (0.97–1.01)	0.36	ref	0.95 (0.84–1.08)	0.44	0.96 (0.85–1.10)	0.57	0.91 (0.79–1.05)	0.20	0.27
**AMD**										
Events, n/N			ref	499/33 649		507/31 266		369/24 269		1736/113 829
Model 1	0.97 (0.96–0.99)	0.009	ref	0.93 (0.81–1.06)	0.26	0.94 (0.82–1.07)	0.35	0.84 (0.73–0.97)	0.021	0.038
Model 2	0.98 (0.96–0.99)	0.010	ref	0.93 (0.81–1.06)	0.27	0.94 (0.82–1.08)	0.336	0.84 (0.73–0.98)	0.025	0.044
Model 3	0.98 (0.96–1.00)	0.015	ref	0.93 (0.81–1.07)	0.30	0.95 (0.83–1.09)	0.44	0.85 (0.73–0.99)	0.035	0.06

AMD – age-related eye disease, BMI – body mass index, MEDLIFE – Mediterranean lifestyle, ref – reference

*Model 1 was adjusted for age, sex, ethnicity, education level, assessment centre, deprivation index, and smoking status. Model 2 was further adjusted from model 1 for total energy intake and BMI group. Model 3 was further adjusted from model 2 for history of hypertension and diabetes.

In the stratified analysis, participants who developed cataract showed no significant interactions with adherence to MEDLIFE concerning age and BMI groups. However, we noted significant interactions in terms of sex and smoking status. Specifically, men were more likely to benefit from higher adherence to MEDLIFE, exhibiting a lower risk of cataract compared to women (*P*-value for interaction = 0.004). The association between adherence to MEDLIFE and cataract risk differed significantly between smokers and non-smokers, with previous and current smokers generally having lower HRs associated with the MEDLIFE index than non-smokers (*P*-value for interaction = 0.019, Table S2 in the **Online Supplementary Document**). We found no interaction effects in the stratified analysis for patients who developed glaucoma or AMD.

### Association between MEDLIFE Blocks and items with risk of age-related eye diseases

A one point increment in the MEDLIFE block 2, *i.e.* ‘Mediterranean dietary habits’ (HR = 0.97; 95% CI = 0.95–0.99)m and block 3, *i.e.* ‘physical activity, rest, social habits and conviviality’ (HR = 0.97; 95% CI = 0.95–0.99) decreased the risk of cataract in the most adjusted model, while block 3 (HR = 0.94; 95% CI = 0.90–0.98) was significantly associated with a lower risk of glaucoma, and block 2 (HR = 0.95; 95% CI = 0.91–0.99) with a lower risk of AMD. However, we did not observe any association between block 1, *i.e.* ‘Mediterranean food consumption’, and the incidence of the three age-related eye diseases (**Table 3**).

**Table 3:** Associations between MEDLIFE blocks and risk of age-related eye diseases in the UK biobank*

	Cataract		Glaucoma		AMD	
	**HR (95%CI)**	***P*-value**	**HR (95%CI)**	***P*-value**	**HR (95%CI)**	***P*-value**
**Block 1: Mediterranean food consumption**						
Model 1	0.99 (0.98–1.01)	0.24	1.01 (0.99–1.04)	0.33	0.99 (0.96–1.02)	0.46
Model 2	1.00 (0.98–1.01)	0.54	1.01 (0.99–1.04)	0.31	0.99 (0.96–1.02)	0.47
Model 3	1.00 (0.99–1.01)	0.65	1.02 (0.99–1.04)	0.29	0.99 (0.96–1.02)	0.51
**Block 2: Mediterranean dietary habits**						
Model 1	0.96 (0.95–0.98)	<0.001	0.99 (0.95–1.03)	0.52	0.95 (0.91–0.98)	0.005
Model 2	0.97 (0.95–0.99)	<0.001	1.00 (0.96–1.03)	0.81	0.95 (0.91–0.99)	0.008
Model 3	0.97 (0.95–0.99)	<0.001	1.00 (0.96–1.04)	0.87	0.95 (0.91–0.99)	0.009
**Block 3: physical activity, rest, social habits, and conviviality**						
Model 1	0.96 (0.95–0.98)	<0.001	0.94 (0.90–0.98)	0.001	0.97 (0.93–1.01)	0.11
Model 2	0.97 (0.95–0.98)	<0.001	0.94 (0.90–0.97)	0.001	0.97 (0.93–1.01)	0.09
Model 3	0.97 (0.95–0.99)	<0.001	0.94 (0.90–0.98)	0.001	0.97 (0.93–1.01)	0.12

AMD – age-related eye disease, BMI – body mass index, MEDLIFE – Mediterranean lifestyle

*Model 1 was adjusted for age, sex, ethnicity, education level, assessment centre, deprivation index, and smoking status. Model 2 was further adjusted from model 1 for total energy intake and BMI group. Model 3 was further adjusted from model 2 for history of hypertension and diabetes.

**Figure 1:**
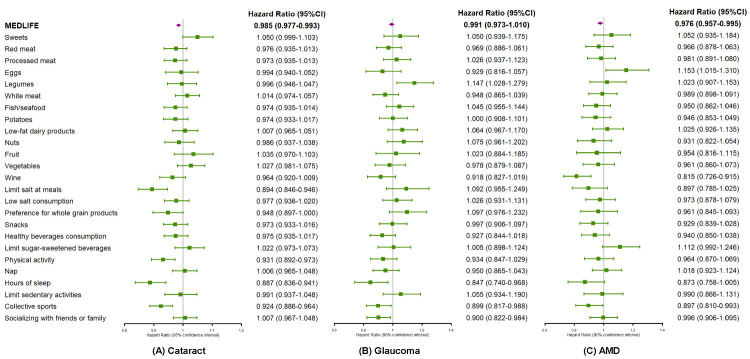
Associations between each MEDLIFE item and risk of age-related eye diseases in the UK Biobank. Each model was adjusted for age, sex, ethnicity, education level, assessment centre, deprivation index, smoking status, total energy intake, BMI group, history of hypertension, and diabetes. The vertical grey lines denote a HR of 1. The purple rhombuses represent the HR for the total MEDLIFE score, and the green squares represent the HR for each MEDLIFE item. Horizontal lines represent the range of the 95% CI. AMD – age-related eye disease, BMI – body mass index, CI – confidence interval, MEDLIFE – Mediterranean lifestyle.

Most of the individual components of the MEDLIFE index exhibited a protective effect against the risk of cataract (**Figure 1**). Limiting salt at meals (HR = 0.89; 95% CI = 0.85–0.95), doing physical activity (HR = 0.93; 95% CI = 0.89–0.97), sleeping 6–8 hours per day (HR = 0.89; 95% CI = 0.84–0.94), and engaging in collective sports (HR = 0.92; 95% CI = 0.89–0.96) were all independently associated with a lower risk of cataract. For glaucoma, sleeping 6–8 hours per day (HR = 0.85; 95% CI = 0.74–0.97), doing collective sports (HR = 0.90; 95% CI = 0.82–0.99), and socialising with friends or family (HR = 0.90; 95% CI = 0.82–0.98) were linked to a reduced risk. Moderate wine consumption (HR = 0.82; 95% CI = 0.73–0.92) and collective sports (HR = 0.90; 95% CI = 0.81–0.99) were identified as protective factors against the onset of AMD.

We arrived at similar results in the sensitivity analyses after excluding participants who completed less than three dietary assessments and after excluding those who were diagnosed with diseases within the first two years of follow-up, when utilising the new MEDLIFE score (27 items) and the new block 1 ‘Mediterranean food consumption’ score (14 items), which incorporated two proxies, and when restricting analyses to white populations (Table S3–9 in the **Online Supplementary Document**).

## DISCUSSION

In this large-scale cohort study, we examined the association between MEDLIFE and the risk of three age-related eye diseases. First, our findings consistently showed that higher adherence to the MEDLIFE was associated with a reduced risk of both cataract and AMD. For each one-point increase in the MEDLIFE score, there was a potential prevention of approximately 1.6% of cataract and 2.1% of AMD cases. By breaking the MEDLIFE into into three distinct blocks, we identified significant associations between block 2 (‘Mediterranean dietary habits’) and risk of cataract and AMD, as well as block 3 (‘physical activity, rest, and social interactions’) and incident cataract and glaucoma. Our findings provide new insights into the importance of MEDLIFE and the specific lifestyle components within its framework and their relation to the risk of age-related eye conditions.

### Associations between MEDLIFE and risk of cataract

Our findings indicated that higher adherence to the MEDLIFE was associated with a lower risk of cataract in a dose-response manner. The beneficial effects are primarily owing to the impacts of the ‘Mediterranean dietary habits’ and ‘physical activity, rest, social habits, and conviviality’ blocks. We discovered no significant relationship between ‘Mediterranean food consumption’ and cataract risk. This is consistent with the findings of a randomised trial which reported a similar incidence of cataract surgery between participants assigned to a Mediterranean diet and those on a low-fat diet over a seven-year follow-up period [33]. However, Shang and colleagues [34] reported a significant inverse correlation between the Alternated Mediterranean diet score and risk of cataract (HR = 0.98; 95% CI = 0.96–0.99) in a UK biobank cohort. The inconsistency between these findings could be attributed to differences in the scoring criteria used to define the Mediterranean diet and variations in the exclusion criteria.

Our findings show that some specific lifestyles, such as reducing salt intake at meals, doing physical activity, sleeping 6–8 hours per day, and engaging in collective sports, are major contributors to the effects of MEDLIFE on cataract risk. This aligns with the results of Bae and colleagues [35], who reported an association between high sodium intake and the development of age-related cataracts, particularly in the elderly, based on the 2008–11 Korea National Health and Nutrition Examination Survey. Another population-based study in Australia found that a high-salt diet doubles the risk of posterior subcapsular cataract in older adults, with those in the highest quintile of sodium intake having twice the incidence compared to those in the lowest quintile [36]. It has been postulated that higher levels of extracellular sodium might hinder the maintenance of lens transparency [37]. Several studies have indicated that physical activity can reduce risk of cataract, potentially by reducing systemic inflammation and oxidative stress involved in the aetiology of age-related cataract [10,38,39]. We further identified that healthy sleeping patterns serve as protective factors against cataract; however, few studies have explored this specific risk. One pooled analysis of 21 studies assessing the relationship between sleep duration and major eye disorders found that short sleep duration is associated with cataract development (odds ratio = 1.20; 95% CI = 1.05–1.36) [40]. A recent cross-sectional study [38] using data from the National Health and Nutrition Examination Survey found unhealthy sleep patterns (<6 hours/day or ≥10 hours/day) to be a risk factor for cataract. An 18-year nationwide population-based study in Taiwan reported a higher risk of cataract in participants with sleep apnoea [41]. All these findings suggest an important role of sleeping and circadian rhythms in eye-care strategies.

### Associations between MEDLIFE and risk of AMD

We also found that higher adherence to MEDLIFE is associated with a decreased frequency of AMD in our sample, with moderate wine consumption and engaging in collective sports acting as protective factors that reach statistical significance. The associations between alcohol consumption and the risk of AMD have been reported in several previous studies, albeit with inconsistencies across results. According to the Beijing Eye study [42], which recruited 4439 adults to investigate the associations between alcohol consumption and ocular disorders, moderate alcohol consumption had no significant effects on the prevalence of AMD. A meta-analysis of seven studies suggested that moderate and heavy, but not light alcohol consumption, could result in a heightened risk of early AMD [43]. However, the Mediterranean alcohol drinking pattern is characterised by moderate alcohol consumption, preferably red wine, mainly with meals and without excess [44]. Relatedly, evidence has suggested that resveratrol and other polyphenols found in red wine, which have antioxidant, anti-inflammatory, and vasorelaxant properties, may improve the microcirculation of the eye, hence preventing the onset or progression of AMD [45,46]. Further investigations are needed on the relationship between Mediterranean alcohol drinking pattern and AMD is warranted.

### Associations between MEDLIFE and risk of glaucoma

Although we did not observe an association between overall MEDLIFE index and risk of glaucoma, we found that higher adherence in the ‘physical activity, rest, social habits and conviviality’ block was associated with lower risk of glaucoma, and the major benefit effects were from doing collective sports, sleeping 6–8 hours per day, and socialising with friends or family. This finding was consistent with the results of prior research [11,47–49], demonstrating that physical activity is associated with a reduced risk of glaucoma development.

We also found that social interaction plays an important role in reducing glaucoma risk. Zhu and colleagues [50] found that social isolation and loneliness increased the risk of glaucoma through biological changes such as increased cortisol secretion and inflammation levels, which led to an elevation in intraocular pressure and accelerated death of retinal ganglion cells. Additionally, normal sleep duration (sleeping 6–8 hours/day) was found to be a protective factor for incident glaucoma in this study, which is possibly due to the influence of melatonin [51,52]. Further studies are necessary to better understand the mechanisms underlying these associations.

### Strength and limitations

To the best of our knowledge, our study is the first large cohort study to explore the associations between Mediterranean lifestyle factors and the risk of age-related eye diseases. However, several limitations should be considered when interpreting its findings. First, although we performed several sensitivity and stratified analyses to test the robustness of our results, bias from reverse causality (*i.e.* poor eyesight leading to reduced physical activity or social engagement) and residual confounding (*i.e.* genetic factors) cannot be completely excluded. Second, the Mediterranean lifestyle factors were assessed using the 24-hour recall questionnaires. Although we calculated mean measures from at least two assessments, this method provides only a limited snapshot of dietary preference and lifestyle choices over a lifetime. Such lifestyle patterns are dynamic and may change over time, potentially introducing bias into our findings. Third, the MEDLIFE index in our study did not account for olive oil or sofrito, which may have led to an underestimation of the effect of the Mediterranean diet. Olive oil is a rich source of monounsaturated fatty acids and contains polyphenols, which play a significant role in its protective effects against AMD [53]. Future studies should explore the association between adherence to the Mediterranean diet, particularly concerning olive oil and sofrito intakes, and the development of age-related eye diseases. Fourth, selection bias might exist since a subgroup is formed when selecting participants with available dietary and lifestyle data. Fifth, the use of hospital in-patient records could underestimate the incident cases of age-related eye diseases, especially for those at the early or mild stage. Furthermore, our analysis only captured the first diagnosis of age-related eye diseases; we were unable to determine the specific disease stage or progression over time, limiting our understanding of the long-term implications of MEDLIFE adherence on the progression of age-related eye diseases. Finally, our findings cannot be generalised to wider populations, as the UK Biobank gathered data among middle-aged UK participants, with limited racial and ethnic diversity.

## CONCLUSIONS

Our findings indicate that a higher adherence to MEDLIFE is significantly associated with a lower risk of cataract and AMD. These findings support the notion that the Mediterranean lifestyle and its specific components may act as a potential behavioural intervention approach for preventing age-related eye diseases. Our findings could inform future clinical trials that could investigate the effects of the MEDLIFE-related interventions on delaying the progression of age-related eye diseases across its various stages.

## Additional material


Online Supplementary Document
