# Supplementary material for: Associations between the Mediterranean lifestyle and incident age-related eye diseases: a longitudinal analysis from the UK Biobank
Source: J Glob Health. 2026 Jan 12;16:04015. doi: 10.7189/jogh.16.04015 (PMC12794434; doi:10.7189/jogh.16.04015)
Supplement: Online Supplementary Document [file jogh-16-04015-s001.pdf]

Supplement to: Li Y, Zhang Y, Wong G, Kam KW, Ho M, Au S, Zhang XJ, Ng MPH, Ip P, Young AL, Pang CP, Tham CC, Chen LJ, Yam JC. Associations between the Mediterranean lifestyle and incident age-related eye diseases: a longitudinal analysis from the UK Biobank. J Glob Health. 2026;16:04015.

Supplementary Materials

Figure S1. Flowchart of the selection of the study population from the UK Biobank.

Table S1. Description of the Mediterranean Lifestyle (MEDLIFE) index, adapted to the UK Biobank.

Table S2. Hazard ratios (95% CI) for the association between MEDLIFE index and age-related eye diseases by subgroups.

Table S3. Hazard ratios (95% CI) for the association between MEDLIFE index and age-related eye diseases in participants who completed three or more dietary questionnaires.

Table S4. Hazard ratios (95% CI) for the association between MEDLIFE index and age-related eye diseases, excluding the first two years of incidence.

Table S5. Hazard ratios (95% CI) for the association between MEDLIFE blocks and age-related eye diseases in participants who completed three or more dietary assessments.

Table S6. Hazard ratios (95% CI) for the association between MEDLIFE blocks and age-related eye diseases, excluding the first two years of incidence.

Table S7. Hazard ratios (95% CI) for the association between the new MEDLIFE index (27 items, using proxies for olive oil and sofrito intakes) and age-related eye diseases.

Table S8. Hazard ratios (95% CI) for the association between new block 1 “Mediterranean food consumption” score (14 items, using proxies for olive oil and sofrito intakes) and age-related eye diseases

Table S9. Hazard ratios (95% CI) for the association between MEDLIFE index and age-related eye diseases in white populations.

Table S10. Outline of JoGH’s Guidelines for Reporting Analyses of Big Data Repositories Open to the Public (GRABDROP) items.

Figure S1. Flowchart of the selection of the study population from the UK Biobank.

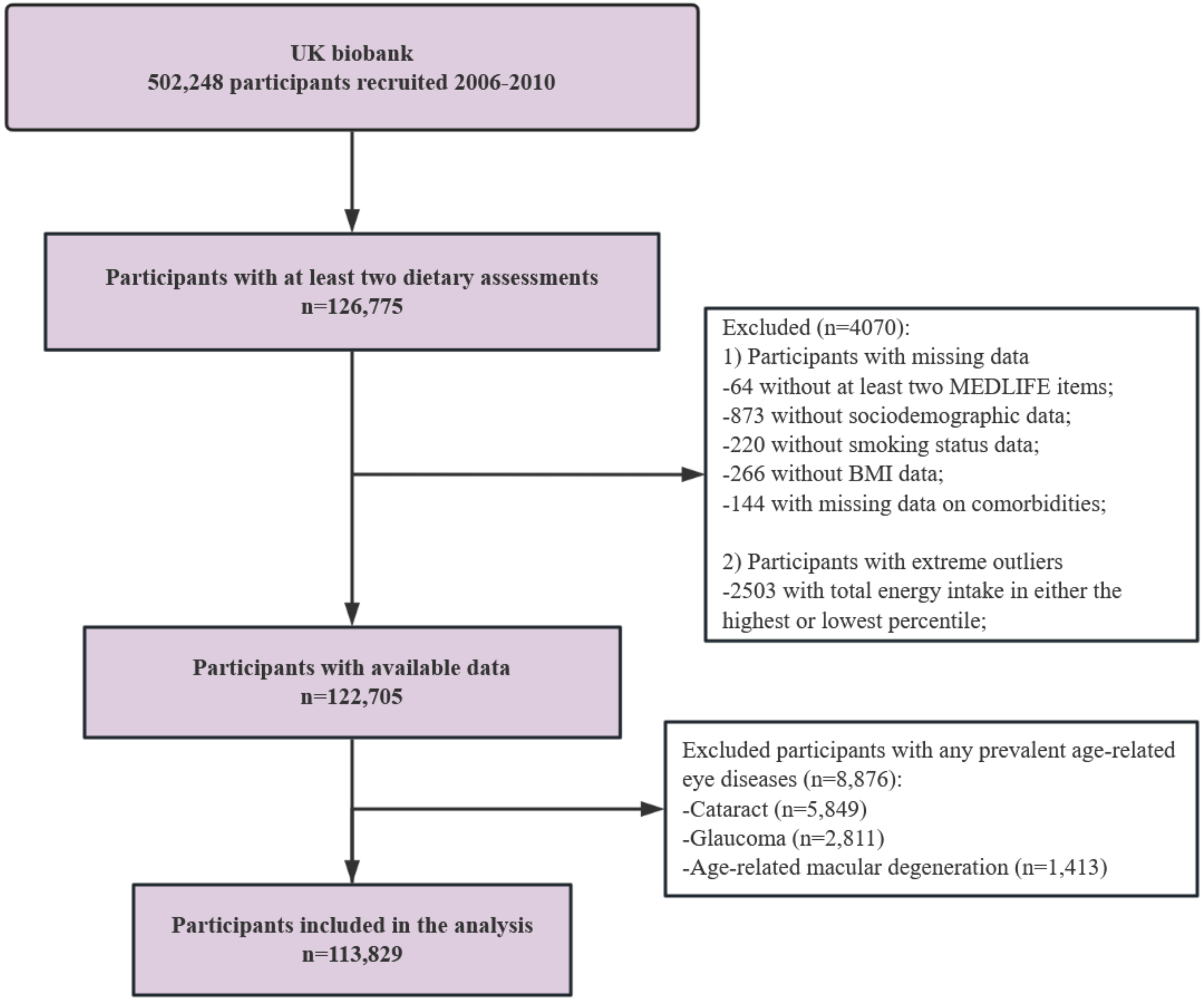

Table S1. Description of the MEDLIFE index, adapted to the UK Biobank.

Index items	Components (serving size)	Criteria for 1 point	No.% scoring 1 point
	Block 1: *Mediterranean food consumption*		
1. Sweets	Cookies, chocolate cookies, pastries, donuts, homemade baked goods, store-bought baked goods, muffins (50 g), chocolates (30 g).	≤ 2 serv/wk	20.4
2. Red meat	Beef, pork, lamb (125 g).	≤ 2 serv/wk	53.0
3. Processed meat	Sausage, soft spicy sausage, bacon, cured ham, cooked ham, hamburger, liver, organ meats, pâté (42.5 g).	≤ 1 serv/wk	46.3
4. Eggs	Eggs (1 unit = 65 g).	≥ 2 to ≤ 4 serv/wk	14.5
5. Legumes	Lentils, beans, chickpeas, peas (150 g cooked).	≥ 2 serv/wk	18.7
6. White meat	Chicken/turkey with skin, chicken/turkey without skin, rabbit (125 g).	≤ 2 serv/wk	60.9
7. Fish/seafood	White fish, fatty fish, codfish, salted or smoked fish, shrimp, octopus, calamari, oysters and shellfish (125 g).	≥ 2 serv/wk	38.2
8. Potatoes	Baked or boiled potatoes (150 g).	≤ 3 serv/wk	37.4
9. Low-fat dairy products	Skim milk, low-fat milk (200ml), low fat yogurt (125 g), fresh soft cheese (50 g).	2 serv/d	30.0
10. Nuts	Almonds, peanuts, hazelnuts, walnuts (30 g).	≥ 3 serv/wk	19.2
11. Sofrito	Sauce of olive oil with onion, pepper, other vegetables (250 g) and tomato (150 g).	> 2/4 ingredients above the median	NA
12. Fruit	Orange, banana, apple, pear, kiwi, mango, avocado, peach, apricot, nectarine, clementine, strawberry, cherries, plums, figs, grapes, watermelon, melon (160 g), dates and dried fruits (30 g).	≥ 3 serv/d	9.3
13. Vegetables	Spinach, cauliflower, broccoli, lettuce, carrot, squash, green beans, eggplant, zucchini, cucumber, pepper, asparagus, gazpacho, garden salad, tomato, other vegetables (236.59 g for green leafy, half for others) *(excluding potatoes).*	≥ 2 serv/d	23.4
14. Olive oil	Olive oil (1 Tbsp = 13.5 g)	≥ 3 serv/d	NA
	Block 2: *Mediterranean* d*ietary habits*		
15. Wine	Red/white wine (1 glass, 100 ml).	Women: > 0 to ≤ 1 serv/d<br>Men: > 0 to ≤ 2 serv/d	25.1
16. Limit salt at meals	Do you add salt to your food? (*After cooking*).	"Never" or "Sometimes"	86.8
17. Low salt consumption	Low sodium-to-potassium ratio and low sodium consumption.	Na/K ratio ≤ 0.57<br>Na ≤ 2.000 mg/d	46.8

18. Preference for whole grain products	Fiber from whole grain cereals.	> 6 g/d fiber from cereals	15.6
19. Snacks	Potato chips, popcorn, or other chips (50 g).	≤ 1 serv/wk	62.1
20. Limit snacking between meals	Do you tend to snack in between meals or before going to bed?	No	NA
21. Healthy beverages consumption	Coffee, decaffeinated coffee (1 cup = 50 ml) or tea (1 cup = 250 ml).	≥ 1 to ≤ 4 serv/d	68.1
22. Limited consumption of sugar-sweetened beverages	Sugar-sweetened beverages + juice (150 ml).	< 1/wk	18.9
Block 3: *Physical activity, rest social habits and conviviality*			
23. Physical activity	Brisk walking, jogging, running, climbing stairs, bicycling, stationary cycling, swimming, dance, aerobic exercise, martial arts, gymnastics, gardening, tennis, soccer, skiing, ice skating, team sports, and other physical activities or sports.	≥ 150 min of moderate, 75 min vigorous, or an equivalent combination	30.7
24. Nap	Napping throughout the week.	"Usually" or "Sometimes" if night-time sleep is adequate	33.7
25. Hours of sleep	Sleeping throughout the week.	6-8 h/d	90.0
26. Limit sedentary activities	Watching TV, using internet or driving.	≤ 2 h/d	18.9
27. Eating in company	Do you have lunch or dinner with friends, family, or others?	Yes	NA
28. Collective sports	Playing soccer, tennis, squash, basketball, or other team sports, running in group, jogging; etc.	≥ 1 h/wk	37.9
29. Socializing with friends or family	Frequency on what you see family (different from whom you live with) or friends (home, church, bars…).	≥ 3 times/wk	45.9

d – day; MEDLIFE – Mediterranean lifestyle; serv – serving; Tbsp – tablespoons; TV – television; wk –week.

Table S2. Hazard ratios (95% CI) for the association between MEDLIFE index and age-related eye diseases by subgroups

	Cataract			Glaucoma			AMD		
	Events/N	HR (95%CI)	*P* interaction	Events/N	HR (95%CI)	*P* interaction	Events/N	HR (95%CI)	*P* interaction
**Sex**									
Male	4050/50020	0.97 (0.96-0.99)***	**0.004**	936/50020	1.00 (0.97-1.02)	0.55	690/50020	0.96 (0.93-0.99)*	0.16
Female	5904/63809	0.99 (0.98-1.00)		1020/63809	1.00 (0.97-1.01)		1046/63809	0.99 (0.96-1.01)	
**Age**									
<60	2894/70155	0.98 (0.97-1.00)*	0.19	717/70155	1.00 (0.98-1.04)	0.51	443/70155	1.01 (0.97-1.05)	0.14
≥60	7060/43674	0.99 (0.98-1.00)*		1239/43674	0.99 (0.96-1.01)		1293/43674	0.98 (0.95-1.00)*	
**Smoking status**									
Non-smoker	5122/65555	0.99 (0.98-1.01)	**0.019**	1047/65555	1.00 (0.98-1.03)	0.53	896/65555	0.98 (0.95-1.00)	0.89
Previous smoker	4206/40437	0.98 (0.97-0.99)**		793/40437	0.97 (0.95-1.00)		741/40437	0.97 (0.95-1.00)	
Current smoker	626/7837	0.94 (0.91-0.97)**		116/7837	1.02 (0.95-1.10)		99/7837	0.99 (0.91-1.07)	
**BMI**									
<25	3561/45416	0.98 (0.97-0.99)**	0.37	747/45416	1.00 (0.97-1.03)	0.67	660/45416	1.00 (0.97-1.03)	0.17
≥25	6393/68413	0.99 (0.98-1.00)*		1209/68413	0.99 (0.97-1.02)		1076/68413	0.97 (0.94-0.99)*	

AMD – age-related eye disease; BMI – body mass index; MEDLIFE – Mediterranean lifestyle.

Model adjusted for age, sex, ethnicity, education level, assessment center, deprivation index, smoking status, total energy intake, BMI group, and history of hypertension and diabetes.

**P*<0.05; ***P*<0.005; ****P*<0.001.

Table S3. Hazard ratios (95% CI) for the association between MEDLIFE index and age-related eye diseases in participants who completed three or more dietary questionnaires

	**Per one point increase**		**Quartile 1 0-7p**	**Quartile 2 8-9p**		**Quartile 3 10-11p**		**Quartile 4 12-25p**		***P*-trend**
	HR (95%CI)	*P* value		HR (95%CI)	*P* value	HR (95%CI)	*P* value	HR (95%CI)	*P* value	
**Cataract**										
Events			1360/16214	1781/21176		1668/19002		1300/14555		6109/70947
Model 1	0.98 (0.97-0.99)	**<0.001**	Reference	0.92 (0.86-0.99)	**0.024**	0.90 (0.84-0.97)	**0.004**	0.90 (0.83-0.97)	**0.005**	**0.005**
Model 2	0.98 (0.97-0.99)	**0.003**	Reference	0.93 (0.87-1.00)	**0.044**	0.92 (0.85-0.98)	**0.017**	0.92 (0.85-1.00)	**0.038**	**0.037**
Model 3	0.99 (0.98-1.00)	**0.007**	Reference	0.93 (0.87-1.00)	0.05	0.92 (0.86-0.99)	**0.028**	0.93 (0.86-1.01)	0.07	0.07
**Glaucoma**										
Events			271/16214	368/21176		345/19002		242/14555		1226/70947
Model 1	0.99 (0.97-1.01)	0.35	Reference	0.98 (0.84-1.15)	0.83	0.99 (0.84-1.16)	0.85	0.89 (0.74-1.07)	0.21	0.25
Model 2	0.99 (0.97-1.01)	0.27	Reference	0.98 (0.84-1.15)	0.79	0.98 (0.83-1.15)	0.78	0.88 (0.74-1.05)	0.16	0.19
Model 3	0.99 (0.97-1.01)	0.32	Reference	0.98 (0.84-1.15)	0.82	0.98 (0.84-1.16)	0.84	0.89 (0.74-1.06)	0.19	0.22
**AMD**										
Events			229/16214	323/21176		296/19002		214/14555		1062/70947
Model 1	0.97 (0.95-0.99)	**0.015**	Reference	0.98 (0.83-1.16)	0.83	0.93 (0.78-1.11)	0.42	0.85 (0.70-1.02)	0.08	0.06
Model 2	0.97 (0.95-0.99)	**0.016**	Reference	0.98 (0.83-1.17)	0.85	0.93 (0.78-1.11)	0.44	0.85 (0.70-1.03)	0.09	0.07
Model 3	0.97 (0.95-1.00)	**0.023**	Reference	0.99 (0.83-1.17)	0.88	0.94 (0.79-1.12)	0.49	0.86 (0.71-1.04)	0.11	0.09

AMD – age-related eye disease; BMI – body mass index; MEDLIFE – Mediterranean lifestyle.

Model 1 adjusted for age, sex, ethnicity, education level, assessment center, deprivation index, and smoking status; Model 2 adjusted for age, sex, ethnicity, education level, assessment center, deprivation index, smoking status, total energy intake and BMI group; Model 3 adjusted for age, sex, ethnicity, education level, assessment center, deprivation index, smoking status, total energy intake, BMI group, and history of hypertension and diabetes.

Table S4. Hazard ratios (95% CI) for the association between MEDLIFE index and age-related eye diseases, excluding the first two years of incidence

	**Per one point increase**		**Quartile 1 0-7p**	**Quartile 2 8-9p**		**Quartile 3 10-11p**		**Quartile 4 12-25p**		***P*-trend**
	HR (95%CI)	*P* value		HR (95%CI)	*P* value	HR (95%CI)	*P* value	HR (95%CI)	*P* value	
**Cataract**										
Events			1924/24304	2627/33151		2562/30793		2041/23976		9154/112224
Model 1	0.98 (0.97-0.99)	**<0.001**	Reference	0.92 (0.87-0.97)	**0.004**	0.90 (0.85-0.95)	**<0.001**	0.89 (0.83-0.95)	**<0.001**	**0.005**
Model 2	0.98 (0.97-0.99)	**<0.001**	Reference	0.93 (0.87-0.98)	**0.012**	0.92 (0.86-0.97)	**0.004**	0.91 (0.86-0.97)	**0.005**	**0.007**
Model 3	0.99 (0.98-0.99)	**0.001**	Reference	0.93 (0.88-0.99)	**0.019**	0.92 (0.87-0.98)	**0.008**	0.92 (0.86-0.98)	**0.013**	**0.017**
**Glaucoma**										
Events			348/24417	494/33305		491/30971		352/24061		1685/112754
Model 1	0.99 (0.97-1.01)	0.24	Reference	0.98 (0.85-1.12)	0.75	1.00 (0.87-1.14)	0.96	0.90 (0.77-1.04)	0.16	0.22
Model 2	0.99 (0.97-1.01)	0.28	Reference	0.98 (0.86-1.13)	0.79	1.00 (0.87-1.15)	0.97	0.90 (0.78-1.05)	0.19	0.26
Model 3	0.99 (0.97-1.01)	0.34	Reference	0.99 (0.86-1.13)	0.84	1.01 (0.88-1.16)	0.90	0.91 (0.78-1.06)	0.23	0.31
**AMD**										
Events			348/24469	473/33352		474/31003		352/24109		1647/112933
Model 1	0.97 (0.96-0.99)	**0.008**	Reference	0.91 (0.79-1.04)	0.18	0.91 (0.79-1.04)	0.18	0.83 (0.72-0.97)	**0.016**	**0.025**
Model 2	0.97 (0.95-0.99)	**0.008**	Reference	0.91 (0.79-1.05)	0.18	0.91 (0.79-1.05)	0.18	0.83 (0.71-0.97)	**0.016**	**0.025**
Model 3	0.97 (0.96-0.99)	**0.011**	Reference	0.91 (0.79-1.05)	0.20	0.92 (0.80-1.05)	0.21	0.84 (0.72-0.98)	**0.022**	**0.034**

AMD – age-related eye disease; BMI – body mass index; MEDLIFE – Mediterranean lifestyle.

Model 1 adjusted for age, sex, ethnicity, education level, assessment center, deprivation index, and smoking status; Model 2 adjusted for age, sex, ethnicity, education level, assessment center, deprivation index, smoking status, total energy intake and BMI group; Model 3 adjusted for age, sex, ethnicity, education level, assessment center, deprivation index, smoking status, total energy intake, BMI group, and history of hypertension and diabetes.

Table S5. Hazard ratios (95% CI) for the association between MEDLIFE blocks and age-related eye diseases in participants who completed three or more dietary assessments

	**Cataract**		**Glaucoma**		**AMD**	
	HR (95%CI)	*P* value	HR (95%CI)	*P* value	HR (95%CI)	*P* value
Events	6109/70947		1226/70947		1062/70947	
**Block 1**						
Model 1	0.99 (0.98-1.01)	0.23	1.01 (0.98-1.05)	0.53	0.99 (0.96-1.03)	0.67
Model 2	1.00 (0.98-1.01)	0.48	1.01 (0.98-1.04)	0.61	0.99 (0.96-1.03)	0.65
Model 3	1.00 (0.98-1.01)	0.56	1.01 (0.98-1.04)	0.59	0.99 (0.96-1.03)	0.71
**Block 2**						
Model 1	0.97 (0.95-0.99)	**0.002**	1.00 (0.95-1.04)	0.83	0.92 (0.88-0.97)	**0.002**
Model 2	0.97 (0.95-0.99)	**0.011**	0.99 (0.95-1.04)	0.77	0.92 (0.88-0.97)	**0.003**
Model 3	0.97 (0.95-1.00)	**0.018**	0.99 (0.95-1.04)	0.82	0.92 (0.88-0.97)	**0.003**
**Block 3**						
Model 1	0.97 (0.95-0.99)	**0.002**	0.94 (0.89-0.98)	**0.008**	0.97 (0.92-1.02)	0.19
Model 2	0.97 (0.95-0.99)	**0.006**	0.94 (0.89-0.98)	**0.007**	0.96 (0.91-1.01)	0.14
Model 3	0.97 (0.95-0.99)	**0.014**	0.94 (0.89-0.98)	**0.009**	0.97 (0.92-1.02)	0.18

AMD – age-related eye disease; BMI – body mass index; MEDLIFE – Mediterranean lifestyle.

Model 1 adjusted for age, sex, ethnicity, education level, assessment center, deprivation index, and smoking status; Model 2 adjusted for age, sex, ethnicity, education level, assessment center, deprivation index, smoking status, total energy intake and BMI group; Model 3 adjusted for age, sex, ethnicity, education level, assessment center, deprivation index, smoking status, total energy intake, BMI group, and history of hypertension and diabetes.

Table S6. Hazard ratios (95% CI) for the association between MEDLIFE blocks and age-related eye diseases, excluding the first two years of incidence

	**Cataract**		**Glaucoma**		**AMD**	
	HR (95%CI)	*P* value	HR (95%CI)	*P* value	HR (95%CI)	*P* value
Events	9154/112224		1685/112754		1647/112933	
**Block 1**						
Model 1	0.99 (0.98-1.01)	0.23	1.01 (0.99-1.04)	0.36	0.99 (0.96-1.01)	0.31
Model 2	1.00 (0.98-1.01)	0.52	1.01 (0.99-1.04)	0.35	0.99 (0.96-1.01)	0.30
Model 3	1.00 (0.98-1.01)	0.62	1.01 (0.99-1.04)	0.34	0.99 (0.96-1.02)	0.33
**Block 2**						
Model 1	0.96 (0.95-0.98)	**<0.001**	0.99 (0.95-1.03)	0.59	0.96 (0.92-1.00)	**0.032**
Model 2	0.97 (0.95-0.99)	**0.001**	1.00 (0.95-1.04)	0.81	0.96 (0.92-1.00)	**0.047**
Model 3	0.97 (0.95-0.99)	**0.001**	1.00 (0.96-1.05)	0.84	0.96 (0.92-1.00)	0.05
**Block 3**						
Model 1	0.97 (0.95-0.98)	**<0.001**	0.94 (0.90-0.97)	**0.001**	0.96 (0.92-1.00)	**0.047**
Model 2	0.97 (0.95-0.99)	**0.001**	0.93 (0.90-0.97)	**0.001**	0.96 (0.92-1.00)	**0.033**
Model 3	0.97 (0.96-0.99)	**0.002**	0.94 (0.90-0.97)	**0.001**	0.96 (0.92-1.00)	**0.045**

AMD – age-related eye disease; BMI – body mass index; MEDLIFE – Mediterranean lifestyle.

Model 1 adjusted for age, sex, ethnicity, education level, assessment center, deprivation index, and smoking status; Model 2 adjusted for age, sex, ethnicity, education level, assessment center, deprivation index, smoking status, total energy intake and BMI group; Model 3 adjusted for age, sex, ethnicity, education level, assessment center, deprivation index, smoking status, total energy intake, BMI group, and history of hypertension and diabetes.

Table S7. Hazard ratios (95% CI) for the association between the new MEDLIFE index (27 items, using proxies for olive oil* and sofrito intakes†) and age-related eye diseases

	**Per one point increase**		**Quartile 1 0-8p**	**Quartile 2 9-10p**		**Quartile 3 11-12p**		**Quartile 4 13-27p**		***P*-trend**
	HR (95%CI)	*P* value		HR (95%CI)	*P* value	HR (95%CI)	*P* value	HR (95%CI)	*P* value	
**Cataract**										
Events			2079/24388	2847/33443		2800/31328		2228/24670		9954/113829
Model 1	0.98 (0.97-0.99)	**<0.001**	Reference	0.92 (0.87-0.97)	**0.003**	0.90 (0.85-0.95)	**<0.001**	0.88 (0.83-0.94)	**<0.001**	**<0.001**
Model 2	0.99 (0.98-0.99)	**<0.001**	Reference	0.93 (0.88-0.98)	**0.009**	0.91 (0.86-0.97)	**0.002**	0.91 (0.85-0.96)	**0.002**	**0.002**
Model 3	0.99 (0.98-0.99)	**<0.001**	Reference	0.93 (0.88-0.99)	**0.015**	0.92 (0.87-0.98)	**0.005**	0.92 (0.86-0.97)	**0.005**	**0.006**
**Glaucoma**										
Events			414/24388	560/33443		561/31328		421/24670		1956/113829
Model 1	0.99 (0.97-1.01)	0.27	Reference	0.93 (0.82-1.06)	0.26	0.95 (0.84-1.08)	0.43	0.89 (0.77-1.02)	0.09	0.15
Model 2	0.99 (0.97-1.01)	0.34	Reference	0.93 (0.82-1.06)	0.30	0.96 (0.84-1.09)	0.52	0.90 (0.78-1.03)	0.12	0.20
Model 3	0.99 (0.98-1.01)	0.41	Reference	0.94 (0.83-1.07)	0.33	0.97 (0.85-1.10)	0.59	0.90 (0.79-1.04)	0.15	0.24
**AMD**										
Events			353/24388	499/33443		512/31328		372/24670		1736/113829
Model 1	0.98 (0.96-1.00)	**0.011**	Reference	0.94 (0.82-1.08)	0.38	0.96 (0.84-1.10)	0.54	0.85 (0.73-0.98)	**0.028**	**0.049**
Model 2	0.98 (0.96-1.00)	**0.013**	Reference	0.94 (0.82-1.08)	0.40	0.96 (0.84-1.10)	0.56	0.85 (0.73-0.99)	**0.033**	0.06
Model 3	0.98 (0.96-1.00)	**0.019**	Reference	0.95 (0.83-1.09)	0.44	0.97 (0.84-1.11)	0.64	0.86 (0.74-1.00)	**0.046**	0.08

AMD – age-related eye disease; BMI – body mass index; MEDLIFE – Mediterranean lifestyle.

*Sofrito intake (item 11) was surrogated by tomato-based sauce consumption. Participants reported tomato-based sauce consumption received a score of 1 point, whereas non-consumers scored 0 points;

†Olive oil intake (item 14) was surrogated by olive oil consumption, derived from “Type of fat/oil used for cooking” in the UK Biobank questionnaire. Participants reported olive oil consumption score 1 point, whereas non-consumers scored 0 points;

Model 1 adjusted for age, sex, ethnicity, education level, assessment center, deprivation index, and smoking status; Model 2 adjusted for age, sex, ethnicity, education level, assessment center, deprivation index, smoking status, total energy intake and BMI group; Model 3 adjusted for age, sex, ethnicity, education level, assessment center, deprivation index, smoking status, total energy intake, BMI group, and history of hypertension and diabetes.

Table S8. Hazard ratios (95% CI) for the association between new block 1 "Mediterranean food consumption" score (14 items, using proxies for olive oil* and sofrito intakes†) and age-related eye diseases

	**Cataract**		**Glaucoma**		**AMD**	
	HR (95%CI)	*P* value	HR (95%CI)	*P* value	HR (95%CI)	*P* value
Events	9954/113829		1956/113829		1736/113829	
Block 1 "Mediterranean food consumption"						
Model 1	0.99 (0.98-1.00)	0.22	1.01 (0.99-1.04)	0.33	0.99 (0.97-1.02)	0.48
Model 2	1.00 (0.99-1.01)	0.54	1.01 (0.99-1.04)	0.33	0.99 (0.97-1.02)	0.47
Model 3	1.00 (0.99-1.01)	0.67	1.01 (0.99-1.04)	0.30	0.99 (0.97-1.02)	0.52

AMD – age-related eye disease; BMI – body mass index; MEDLIFE – Mediterranean lifestyle.

*Sofrito intake (item 11) was surrogated by tomato-based sauce consumption. Participants reported tomato-based sauce consumption received a score of 1 point, whereas non-consumers scored 0 points.

†Olive oil intake (item 14) was surrogated by olive oil consumption, derived from "Type of fat/oil used for cooking" in the UK Biobank questionnaire. Participants reported olive oil consumption score 1 point, whereas non-consumers scored 0 points.

Model 1 adjusted for age, sex, ethnicity, education level, assessment center, deprivation index, and smoking status; Model 2 adjusted for age, sex, ethnicity, education level, assessment center, deprivation index, smoking status, total energy intake and BMI group; Model 3 adjusted for age, sex, ethnicity, education level, assessment center, deprivation index, smoking status, total energy intake, BMI group, and history of hypertension and diabetes.

Table S9. Hazard ratios (95% CI) for the association between MEDLIFE index and age-related eye diseases in white populations

	**Per one point increase**		**Quartile 1 0-7p**	**Quartile 2 8-9p**		**Quartile 3 10-11p**		**Quartile 4 12-25p**		***P*-trend**
	HR (95%CI)	*P* value		HR (95%CI)	*P* value	HR (95%CI)	*P* value	HR (95%CI)	*P* value	
**Cataract**										
Events			2054/23984	2772/32714		2709/30263		2126/23545		9661/110506
Model 1	0.98 (0.97-0.99)	**<0.001**	Reference	0.91 (0.86-0.96)	**0.001**	0.89 (0.84-0.95)	**<0.001**	0.88 (0.82-0.93)	**<0.001**	**<0.001**
Model 2	0.98 (0.98-0.99)	**<0.001**	Reference	0.92 (0.87-0.97)	**0.004**	0.91 (0.86-0.96)	**0.001**	0.90 (0.85-0.96)	**0.001**	**0.002**
Model 3	0.98 (0.98-0.99)	**<0.001**	Reference	0.92 (0.87-0.98)	**0.007**	0.92 (0.86-0.97)	**0.003**	0.91 (0.86-0.97)	**0.003**	**0.005**
**Glaucoma**										
Events			405/23984	550/32714		531/30263		407/23545		1893/110506
Model 1	0.99 (0.97-1.01)	0.24	Reference	0.94 (0.83-1.07)	0.33	0.93 (0.82-1.06)	0.30	0.91 (0.79-1.04)	0.16	0.19
Model 2	0.99 (0.97-1.01)	0.31	Reference	0.94 (0.83-1.07)	0.37	0.94 (0.83-1.07)	0.37	0.91 (0.79-1.05)	0.21	0.24
Model 3	0.99 (0.97-1.01)	0.36	Reference	0.95 (0.83-1.08)	0.41	0.95 (0.83-1.08)	0.42	0.92 (0.80-1.06)	0.25	0.29
**AMD**										
Events			357/23984	486/32714		493/30263		358/23545		1694/110506
Model 1	0.97 (0.96-0.99)	**0.007**	Reference	0.91 (0.79-1.04)	0.18	0.93 (0.81-1.06)	0.26	0.83 (0.72-0.96)	**0.014**	**0.027**
Model 2	0.97 (0.96-0.99)	**0.008**	Reference	0.91 (0.80-1.05)	0.19	0.93 (0.81-1.06)	0.28	0.83 (0.72-0.97)	**0.017**	**0.032**
Model 3	0.98 (0.96-1.00)	**0.012**	Reference	0.92 (0.80-1.05)	0.21	0.93 (0.81-1.07)	0.33	0.84 (0.72-0.98)	**0.023**	**0.044**

AMD – age-related eye disease; BMI – body mass index; MEDLIFE – Mediterranean lifestyle.

Model 1 adjusted for age, sex, ethnicity, education level, assessment center, deprivation index, and smoking status; Model 2 adjusted for age, sex, ethnicity, education level, assessment center, deprivation index, smoking status, total energy intake and BMI group; Model 3 adjusted for age, sex, ethnicity, education level, assessment center, deprivation index, smoking status, total energy intake, BMI group, and history of hypertension and diabetes.

Table S10. Outline of JoGH's Guidelines for Reporting Analyses of Big Data Repositories Open to the Public (GRABDROP) items.

1. Please list all papers published by each co-author in previous three years that were based on secondary analysis of a big data repository	The following studies based on UK Biobank data were conducted by co-authors:<br>1. Gao Y, Wang Y, Zhang Y, Yu J, Xu J, Kam KW, Ho M, Young AL, Pang CP, Tham CC, Yam JC, Chen LJ. Accelerated Biological Aging and Genetic Pleiotropy in Age-Related Eye Diseases: A Population-Based Cohort and Integrative Genetic Analysis. Invest Ophthalmol Vis Sci. 2025 Nov 3;66(14):14.<br>2. Yu J, Zhang Y, Ho M, Kam KW, Young AL, Pang CP, Tham CC, Yam JC, Chen LJ. Association of leucocyte telomere length with incident age-related macular degeneration: a prospective UK Biobank Study. Br J Ophthalmol. 2025 Sep 4:bjo-2025-327492.<br>3. Peng Y, Zhang Y, Kam KW, Ho M, Au SCL, Zhang X, Ng MPH, Ip P, Young AL, Pang CP, Tham CC, Chen LJ, Yam JC. Association of serum 25-hydroxyvitamin D with cataract: a cross-sectional and longitudinal analysis of the UK Biobank. Br J Ophthalmol. 2025 Dec 15;110(1):39-45.<br>4. Peng Y, Zhang Y, Kam KW, Ho M, Au S, Zhang X, Ng MP, Ip P, Young A, Pang CP, Tham CC, Chen LJ, Yam JC. Sleep, physical activity, sedentary behavior, and risk of cataract: a cross-sectional and prospective study from UK Biobank. BMC Med. 2025 Aug 8;23(1):466.<br>5. Zaabaar E, Zhang Y, Kam KW, Li Y, Zhang XJ, Ho M, Liu D, Ng MP, Ip P, Young A, Pang CP, Tham CC, Kwan MP, Chen LJ, Yam JC. Association of residential air pollution with visual impairment in adults: The UK Biobank study. Asia Pac J Ophthalmol (Phila). 2025 Nov-Dec;14(6):100209.<br>6. Yu J, Zhang Y, Pang CP, Tham CC, Yam JC, Chen LJ. Association between leukocyte telomere length and incident glaucoma: A prospective UK biobank study. Eye (Lond). 2025 Aug;39(11):2176-2182.<br>7. Peng Y, Zhang Y, Kam KW, Wong G, Ho M, Sezto S, Au S, Zhang X, Ng MPH, Ip P, Young A, Pang CP, Tham CC, Chen LJ, Yam JC. Associations of Cardiovascular Health and New-Onset Age-Related Macular Diseases From UK Biobank. Invest Ophthalmol Vis Sci. 2025 Apr 1;66(4):63.<br>8. Li Y, Zhang Y, Kam KW, Chan P, Liu D, Zaabaar E, Zhang XJ, Ho M, Ng MP, Ip P, Young A, Pang CP, Tham CC, Kwan MP, Chen LJ, Yam JC. Associations of long-term joint exposure to multiple ambient air pollutants with the incidence of age-related eye diseases. Ecotoxicol Environ Saf. 2025 Apr 1;294:118052.<br>9. Yu J, Zhang Y, Kam KW, Ho M, Young AL, Pang CP, Tham CC, Yam JC, Chen LJ. Lung Function as a Biomarker for Glaucoma: The UK Biobank Study. Invest Ophthalmol Vis Sci. 2025 Feb 3;66(2):48.<br>10. Yu J, Zhang Y, Ho M, Zhang XJ, Kam KW, Young AL, Pang CP, Tham CC, Yam JC, Chen LJ. Association of Metabolomics With Incidence of Age-Related Macular Degeneration: The UK Biobank Study. Invest Ophthalmol Vis Sci. 2024 Dec 2;65(14):43.

2. Please explain the key elements of your study design and the use of the available datasets that make your study an original scientific contribution	This study utilized data from the UK Biobank to investigate the association between the Mediterranean lifestyle (MEDLIFE) and the incidence of three major age-related eye diseases: cataract, glaucoma, and age-related macular degeneration (AMD). To the best of our knowledge, this is the first large cohort study to explore the holistic effect of Mediterranean lifestyle factors on the risk of age-related eye diseases. By employing rigorous statistical methods, including cox proportional hazards models validated by Schoenfeld global tests, alongside a series of sensitivity and stratified analyses, we established robust longitudinal associations. Our findings provide new insights into the importance of MEDLIFE and the specific lifestyle components within its framework and their relation to the risk of age-related eye conditions.
3. Please list all publications that addressed similar research questions in the same dataset and indicate where you cited them in your paper	One relevant publication was listed as followed: Shang X, Liu J, Zhu Z, Zhang X, Huang Y, Liu S, Wang W, Zhang X, Tang S, Hu Y, Yu H, Ge Z, He M. Healthy dietary patterns and the risk of individual chronic diseases in community-dwelling adults. Nat Commun. 2023 Oct 23;14(1):6704., cited as reference 34 in our paper.
4. Please explain how you addressed multiple testing through an appropriately rigorous statistical threshold and indicate this in the methods section	Given the distinct etiologies of cataract, glaucoma, and AMD, we analyzed these outcomes as independent hypotheses and maintained a significance threshold of $P < 0.05$. While we did not apply a multiple testing correction, we addressed the risk of false-positive results by conducting rigorous sensitivity analyses. These additional tests consistently supported our main findings, suggesting that the observed associations are robust.
5. Please declare to what extent have AI chatbots been used in developing your paper and to which parts of the paper did they contribute	We declare that AI chatbots were not used at any stage in the development of this manuscript.

Adapted from: Rudan I, Song P, Adeloye D, Campbell H. Journal of Global Health’s Guidelines for Reporting Analyses of Big Dat a Repositories Open to the Public (GRABDROP): preventing ‘paper mills’, duplicate publications, misuse of statistical inference, and inappropriate use of artificial intelligence. J Glob Health. 2025;15:01004.
